# Co-Evolution of Primate SAMHD1 and Lentivirus Vpx Leads to the Loss of the *vpx* Gene in HIV-1 Ancestor

**DOI:** 10.1371/journal.pone.0037477

**Published:** 2012-05-04

**Authors:** Chiyu Zhang, Suresh de Silva, Jian-Hua Wang, Li Wu

**Affiliations:** 1 Institute of Life Sciences, Jiangsu University, Zhenjiang, Jiangsu, China; 2 Department of Veterinary Bioscience, Center for Retrovirus Research, The Ohio State University, Columbus, Ohio, United States of America; 3 Pathogen Diagnostic Center, Institut Pasteur of Shanghai, Shanghai Institutes for Biological Sciences, Chinese Academy of Sciences, Shanghai, China; 4 Key Laboratory of Molecular Virology and Immunology, Institut Pasteur of Shanghai, Shanghai Institutes for Biological Sciences, Chinese Academy of Sciences, Shanghai, China; 5 Department of Microbial Infection and Immunity, The Ohio State University, Columbus, Ohio, United States of America; The University of Hong Kong, Hong Kong

## Abstract

Cross-species transmission and adaptation of simian immunodeficiency viruses (SIVs) to humans have given rise to human immunodeficiency viruses (HIVs). HIV type 1 (HIV-1) and type 2 (HIV-2) were derived from SIVs that infected chimpanzee (SIVcpz) and sooty mangabey (SIVsm), respectively. The HIV-1 restriction factor SAMHD1 inhibits HIV-1 infection in human myeloid cells and can be counteracted by the Vpx protein of HIV-2 and the SIVsm lineage. However, HIV-1 and its ancestor SIVcpz do not encode a Vpx protein and HIV-1 has not evolved a mechanism to overcome SAMHD1-mediated restriction. Here we show that the co-evolution of primate SAMHD1 and lentivirus Vpx leads to the loss of the *vpx* gene in SIVcpz and HIV-1. We found evidence for positive selection of SAMHD1 in orangutan, gibbon, rhesus macaque, and marmoset, but not in human, chimpanzee and gorilla that are natural hosts of Vpx-negative HIV-1, SIVcpz and SIVgor, respectively, indicating that *vpx* drives the evolution of primate SAMHD1. Ancestral host state reconstruction and temporal dynamic analyses suggest that the most recent common ancestor of SIVrcm, SIVmnd, SIVcpz, SIVgor and HIV-1 was a SIV that had a *vpx* gene; however, the *vpx* gene of SIVcpz was lost approximately 3643 to 2969 years ago during the infection of chimpanzees. Thus, HIV-1 could not inherit the lost *vpx* gene from its ancestor SIVcpz. The lack of Vpx in HIV-1 results in restricted infection in myeloid cells that are important for antiviral immunity, which could contribute to the AIDS pandemic by escaping the immune responses.

## Introduction

Cross-species transmission and adaptation of SIVs to humans have given rise to HIV-1 and HIV-2, which were derived from SIVs that infected chimpanzee (SIVcpz) and sooty mangabey (SIVsm), respectively. HIVs and SIVs encode several proteins to counteract host restriction factors that block viral infection. For example, HIV-1 Vif and Vpu (or Nef of SIV and the envelope protein of HIV-2) counteract the host antiviral restriction factors APOBEC3G and Tetherin, respectively [1], [2]. The cellular protein SAMHD1 is a human myeloid-cell-specific HIV-1 restriction factor that can be counteracted by the Vpx protein of HIV-2 and the SIVsm lineage [3], [4]. However, HIV-1 and its ancestor SIVcpz do not encode Vpx and HIV-1 has not evolved a mechanism to overcome SAMHD1-mediated restriction. This raises the question whether co-evolution of primate SAMHD1 and lentiviruse Vpx leads to the loss of *vpx* in SIVcpz and HIV-1.

SAMHD1 is a dGTP-regulated triphosphohydrolase that can degrade dNTPs [5], [6] and mediate HIV-1 restriction by decreasing the dNTP pool concentration in the cell [7]. SAMHD1 contains a sterile alpha motif (SAM) domain and a HD domain that has a highly conserved motif with two His (H) and two Asp (D) residues. The SAM domain is involved in protein-protein and/or protein-RNA interactions [8] and the phosphohydrolase activity of the HD domain is crucial for HIV-1 restriction of SAMHD1 [9]. Interestingly, Vpx of HIV-2 and SIVsm can induce proteolytic degradation of SAMHD1 through the CUL4A/DCAF1 E3 ubiquitin ligase [3], [4], which relieves SAMHD1-mediated viral restriction in human myeloid cells. The lack of Vpx in HIV-1 appears to benefit HIV-1 as a fortuitous strategy for viral escape from immune surveillance initiated by the infected myeloid cells [10], [11].

Coevolutionary arms races between host restriction factors and viral countermeasures result in constant natural selection for co-adaptation to each other by rapid amino acid substitutions in both proteins [12]. Two recent studies reported positive selection of primate SAMHD1, while different selection residues in SAMHD1 were identified in these studies [13], [14]. Lim and colleagues suggested that primate lentiviral Vpr has evolved a new function to degrade primate SAMHD1 before the presence of a separate *vpx* gene, thereby initiating an evolutionary arms race with SAMHD1 [13]. In contrast, Laguette *et al.* suggested that SMAHD1 antagonism have appeared simultaneously with or close to the birth of *vpr* [14]. This discrepancy may be due to different experimental approaches and evolutionary analyses, which remains to be confirmed. Moreover, Laguette *et al.* demonstrated that SAMHD1-mediated HIV-1 restriction is evolutionarily maintained and antagonism of SAMHD1 by lentiviral Vpx in a species-specific manner [14]. However, neither of these studies extensively analyzed positive selection of Vpx and its evolution, which should be a critical consideration in understanding co-evolution of primate SAMHD1 and lentiviral Vpx.

In the present study, we confirmed through evolutionary analyses of primate SAMHD1 sequences, that positive selection acts on SAMHD1 of orangutan, gibbon, rhesus macaque, and marmoset, but not on SAMHD1 of human, chimpanzee and gorilla that are the natural hosts of Vpx-negative HIV-1, SIVcpz and SIVgor, respectively. Our analyses indicate that the most recent common ancestor (MRCA) of Vpx-positive SIVrcm and SIVmnd [SIV infecting red-capped mangabeys (*C. torquatus*) and mandrills (*M. sphinx*), respectively], and Vpx-negative SIVcpz, SIVgor and HIV-1 traces back to a SIV that had *vpx* and infected chimpanzees about 3643 years ago. We also performed a phylogenetic analysis of *vpx* genes from SIV and HIV-2. These results indicate that the *vpx* gene was lost during the long-term evolution of SIV among chimpanzees, which is likely due to the genetic conflict between the restriction factor SAMHD1 and the viral antagonist Vpx. Our study provided new insights into co-evolution of primate SAMHD1 and lentivirus Vpx.

## Results

### Phylogeny of the primate SAMHD1 gene sequences

**Figure 1:**
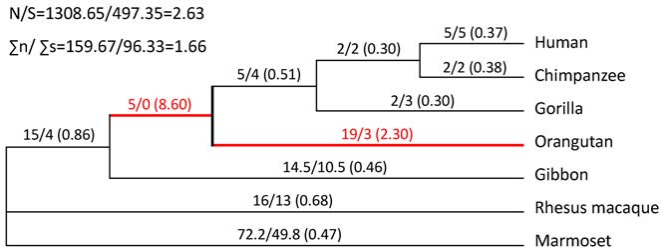
Phylogeny and positive selection of primate *SAMHD1* genes. The actual numbers of n/s changes and ω values (dN/dS, in parentheses) are shown above each branch. N and S are the potential numbers of non-synonymous and synonymous sites, respectively. The thick red lines represent the branches under positive selection.

To analyze phylogeny of the primate *SAMHD1* sequences, we acquired the sequences of human, gorilla, orangutan, gibbon, rhesus macaque and marmoset from GenBank and other genome assembly databases. Due to the incomplete chimpanzee *SAMHD1* sequence in the database, we sequenced *SAMHD1* cDNA derived from RNA of chimpanzee liver, lymph node, and B cells. We constructed Bayesian and maximum likelihood (ML) phylogenetic trees based on the protein coding sequences of the seven primate *SAMHD1* genes. Both trees show identical topologies and the Bayesian tree is shown in Fig. 1. The relationships of seven primate *SAMHD1* genes are consistent with the known species phylogeny, and five hominid species form a monophyletic group (Fig. 1).

### Positive selection on primate SAMHD1

**Figure 2:**
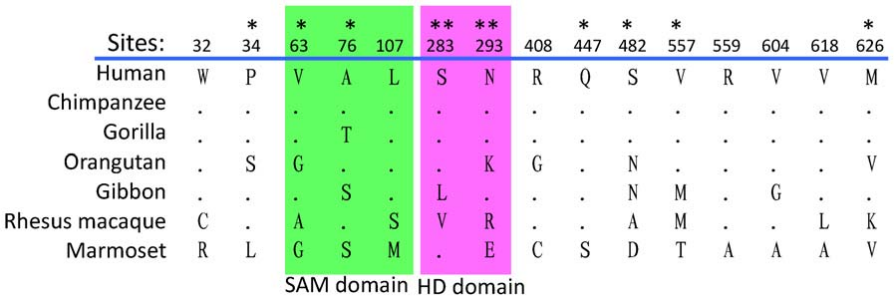
Positively selected residues in primate SAMHD1 proteins. The black small dots indicate identical residues compared to the human SAMHD1 sequence. Positive selected sites were identified with posterior probabilities >0.90. One and two asterisks indicate posterior probabilities ≥0.95 and 0.99, respectively. The green and pink shadows indicate the SAM and HD domains, respectively.

To examine whether positive selection drives the evolution of the primate *SAMHD1* gene, we first calculated the non-synonymous (dN) and synonymous (dS) distances between each pair of the sequences. Only one of 21 pairwise comparisons exhibited slightly higher dN (0.028) than dS (0.027) (Fig. S1), suggesting no significant positive selection. Because positive selection usually affects only a few residues in a protein, we used the site-specific model in the PAML package to detect positive selection and to identify positively selected sites (PSS). The results show that two selection models (M2a and M8) fit the data significantly better than the null models without selection, and 11.3–11.5% amino acid sites of SAMHD1 appear to be under positive selection (ω: 3.95–4.00) (Table 1). Using the M8 model, 15 sites were identified to be under positive selection (ω>1) at the level of posterior probability (p)≥0.90 among all seven primate species analyzed (Fig. 2 and Table 1).

**Table 1:** Phylogenetic analysis by maximum likelihood estimation of the primate SAMHD1 genes.

Dataset	Model code	lnL	Estimates of parameters	2Δl	PSS
**All 7 primate species (see ** **Fig. 1** **)**	M1a (Nearly Neutral)	−3777.65	p0 = 0.57163 p1 = 0.42837 ω0 = 0.00 ω1 = 1.00	22.21 P<0.01	Not allowed
	M2a (Positive Selection)	−3766.54	p0 = 0.61742 p1 = 0.27008 p2 = 0.11250 ω0 = 0.00000 ω1 = 1.00 ω2 = 4.00		34P, 63V*, 76A, 283S*, 293N*, 447Q, 482S*, 626M
	M7 (beta)	−3778.69	p = 0.00503 q = 0.00500	24.30 P<0.01	
	M8 (beta&w>1)	−3766.54	p0 = 0.88519 p = 0.00500 q = 0.01092 (p1 = 0.11481) ω = 3.95		32W, 34P*, 63V* 76A*, 107L, 283S**, 293N**, 408R, 447Q*, 482S*, 557V*, 559R, 604V, 618V, 626M*
**Three primate species: human, chimpanzee and gorilla**	M1a (Nearly Neutral)	−2680.82	p0 = 0.99999 p1 = 0.00001 ω0 = 0.38 ω1 = 1.00	0 P>0.05	Not allowed
	M2a (Positive Selection)	−2680.82	p0 = 1.00000 p1 = 0.00000 p2 = 0.00000 ω0 = 0.38 ω1 = 1.00 ω2 = 1.00		None
	M7 (beta)	−2680.82	p = 61.12811 q = 99.00000	0 P>0.05	Not allowed
	M8 (beta&w>1)	−2680.82	p0 = 0.99999 p = 61.12626 q = 99.00000 (p1 = 0.00001) ω = 1.00		None
**Four primate species: orangutan, gibbon, rhesus macaque, and marmoset**	M1a (Nearly Neutral)	−3523.48	p0 = 0.55026 p1 = 0.44974 ω0 = 0.00 ω1 = 1.00	19.57 P<0.01	Not allowed
	M2a (Positive Selection)	−3513.69	p0 = 0.83059 p1 = 0.00000 p2 = 0.16941 ω0 = 0.16398 ω1 = 1.00 ω2 = 3.72		34P, 283S*, 293N, 447Q, 626M
	M7 (beta)	−3523.97	p = 0.00500 q = 0.00746	20.55 P<0.01	Not allowed
	M8 (beta&w>1)	−3513.69	p0 = 0.83094 p = 19.56472 q = 98.97297 (p1 = 0.16906) ω = 3.72		32W, 34P*, 63V, 283S**, 293N*, 447Q*, 482S, 557V, 626M*

lnL, log-likelihood value. 2Δl, the likelihood ratio test statistics (2 delta lambda statistics). The P values represent a level of significance with a χ2 distribution and degrees of freedom = 2 (M1a vs. M2a and M7 vs. M8). Positively selected sites (PSS) were identified with posterior probability (p)≥0.90. One asterisk indicates posterior probability p≥0.95 and two asterisks p≥0.99. The null models do not allow for a site with ω>1. The amino acid sequence of human SAMHD1 was used as the reference standard. The results from M3 (discrete) are not shown since M3 can overestimate the number of positively selected sites.

Amino acid changes in positive selection contain conservative and radical substitutions [15]. The radical substitutions result in a change in a certain physicochemical property (e.g. the charge, polarity, and polarity and volume) of the amino acid and thereby affect the function of protein. In most cases, positive selection results in radical non-synonymous substitutions [15]. To investigate whether this is the case in primate SAMHD1, we estimated radical and conservative non-synonymous (n) substitutions on each branch of the tree (Table 2). The radical n substitution rates (Σr/R) in charge (1.99 vs. 0.76, P<0.0001, chi-square test) and polarity (3.05 vs. 0.56, P<0.0001, chi-square test) are significantly higher than the conservative substitution rate (Σc/C) (Table 2). These results strongly suggest that the positive selection favors alterations of amino acid charge and polarity in SAMHD1 evolution. However, the radical n substitution rate in polarity and volume is significantly smaller than the conservative substitution rate (0.67 vs. 2.65, P<0.0001, chi-square test) (Table 2), indicating that positive selection of SAMHD1 does not favor alterations of amino acid polarity and volume.

**Table 2:** Numbers of conservative and radical non-synonymous substitutions on the primate branches.

	R^a^	C^a^	Σr^b^	Σc^b^	Σr/R^c^	Σc/C^c^
**Charge**	330.96	453.71	658.84	344	1.99	0.76
**Polarity**	225.76	558.90	687.84	315	3.05	0.56
**Polarity and volume**	541.29	243.37	358.84	644	0.67	2.65

a The potential numbers of radical non-synonymous substitutions and conservative non-synonymous substitutions.

b The total numbers of radical and conservative non-synonymous substitutions on all branches.

c The total radical and conservative non-synonymous substitution ratios of all branches.

### Different selective pressures on primate lineages

Although positive selection was detected on the primate *SAMHD1* gene, low average ω (dN/dS) value (0.61) of seven primate species suggests that different lineages might experience various selective pressures. To address this question, we counted the numbers of non-synonymous (n) and synonymous (s) substitutions on each tree branch (Fig. 1). The sums of n and s for all branches were 159.7 and 96.3, respectively. The potential numbers of non-synonymous (N) and synonymous (S) sites are 1308.7 and 497.4, respectively. As a whole, the Σn/Σs ratio (1.66) is significantly smaller than the N/S ratio (2.63) (P = 0.0011, chi-square test), consistent with low average ω value. However, the branch leading to orangutan and its ancestral branch shared with human, chimpanzee and gorilla have substantially higher n/s ratios (6.33 and ∞ with P = 0.108 and 0.201, respectively, Fisher's exact test) compared with the N/S ratio (2.63) (Fig. 1). The free-ratio model in the PAML package also shows that both branches have substantially higher ω values (2.30 and 8.60, respectively). When both branches were taken as a whole, the n/s ratio (8.00) is significantly higher than the N/S ratio (P = 0.039, Fisher's exact test). These results might suggest some special evolutionary events driving the evolution of orangutan SAMHD1. Of the 19 amino acid changes fixed by orangutan SAMHD1, five were also detected under positive selection by the site-specific model (Fig. S2).

Human, chimpanzee and gorilla are the natural hosts of Vpx-negative HIV-1, SIVcpz and SIVgor, respectively [16]. Given that positive selection acting on the primate SAMHD1 has most likely been driven by Vpx, there should be no positive selection on SAMHD1 from these three primate species. As expected, the site-specific model shows that the two null models (M1a and M7) are not rejected (P>0.05, likelihood ratio test), and there was no site that could be detected under positive selection at the level of p≥0.90 (Table 1). These results indicate that no positive selection acts on SAMHD1 from human, chimpanzee and gorilla, and imply that positive selection detected in primate SAMHD1 might be present in other primate species. Indeed, when the same analysis was performed using *SAMHD1* genes from orangutan, rhesus macaque, gibbon, and marmoset, we detected a significant positive selection signal and identified 9 PSS by the selective model M8 (Table 1).

### Phylogeny of SIVs and HIVs

HIV-2 and some SIV lineages (referred to as SIV(Vpx+)) contain Vpx that can degrade human SAMHD1 [3], [4], whereas HIV-1 and certain SIV strains (e.g. SIVcpz and SIVgor, referred to as SIV(Vpx−)) lack *vpx*. In order to understand the evolutionary basis of HIV-1 and SIV(Vpx−) lacking *vpx* gene, we retrieved all available genomic sequences of SIV, HIV-2, and the subtype reference sequences of HIV-1 group M and reconstructed their phylogenetic relationship. We focused on the M group of HIV-1 since it includes more than 95% of the global HIV-1 isolates [17]. The ML tree was constructed based on the *pol* gene sequences since *pol* is the most highly conserved gene in retroviruses and is able to fully reflect the evolutionary relationship of retroviruses.

**Figure 3:**
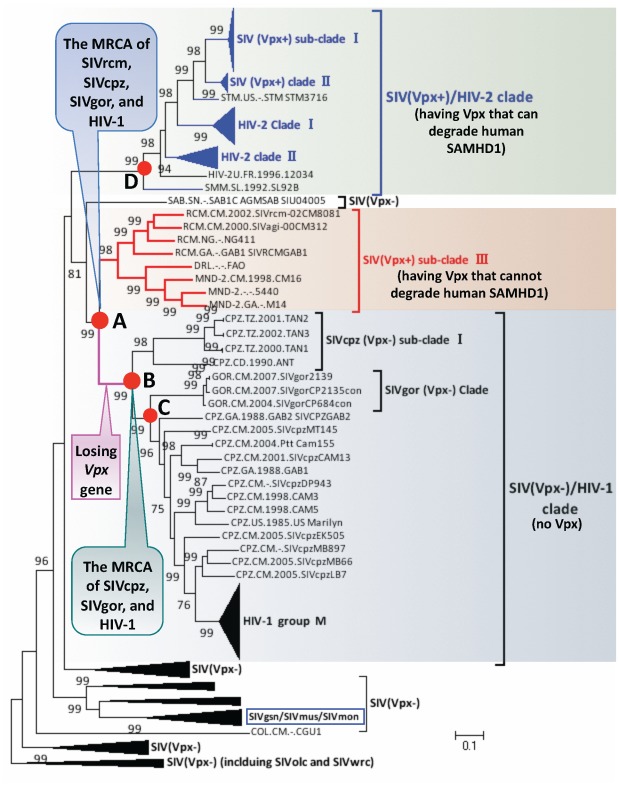
Phylogenetic tree of *pol* genes from primate lentiviruses. Because the Vpx of two SIVrcm isolates from Nigeria and Gabon cannot degrade human SAMHD1 [3], we predicted that Vpx from other SIVs in SIV(Vpx+) sub-clade III may not be able to degrade human SAMHD1. The red solid nodes on the trees represent the most recent common ancestors (MRCA) of corresponding virus strains. The thick pink branch indicates the occurrence of *vpx* gene loss. Only bootstrap values of >75 are shown at the corresponding nodes. This tree was derived from the sequence analysis of 182 primate lentivirus strains/isolates.

In the phylogenetic tree (Fig. 3), SIV(Vpx+) are divided into three sub-clades (I to III), and HIV-2 into two sub-clades (I and II). Of the three SIV(Vpx+) sub-clades, two (I to II) clearly cluster with two HIV-2 sub-clades, forming a clade of SIV(Vpx+)/HIV-2 (bootstrap value: 99%) (Fig. 3), suggesting that these viruses share a common origin. A similar topology was observed in the phylogenetic tree of the *vpx* genes from SIV and HIV-2 (Fig. S3). Hence, their MRCA should contain a functional Vpx to degrade human SAMHD1 since the Vpx from this clade have similar protein sequences (Fig. S4). Furthermore, Vpx of SIVmac-251 and HIV-2 ROD from this clade have been demonstrated to degrade human SAMHD1 [3], [4]. The sequences from HIV-1 group M form an independent group (Fig. 3). SIVcpz(Vpx−), SIVgor(Vpx−) and HIV-1 group M cluster together to form a clade of SIV(Vpx−)/HIV-1 (bootstrap value: 99%) (Fig. 3), suggesting that they share a MRCA at node B. SIVgor(Vpx−) forms an independent cluster. SIVgor(Vpx−) has been demonstrated to evolve into HIV-1 groups O and/or P via cross-species transmission to humans [18]. Interestingly, SIVcpz(Vpx−) sequences do not cluster together to form a monophyletic group (Fig. 3), indicating the presence of various SIVcpz(Vpx−) lineages. A few SIVcpz(Vpx−) cluster together to form a sub-clade. Other SIVcpz(Vpx−) are located between SIVgor(Vpx−) and HIV-1 group M, which is at the most interior node, suggesting a MRCA at node C (Fig. 3).

Because SIVcpz/gor and HIV-1 group M do not have a *vpx* gene, the MRCAs at nodes B and C should not have *vpx*. The SIV(Vpx−)/HIV-1 clade further clusters with the SIV(Vpx+) sub-clade III that contains SIVrcm/mnd(Vpx+) strains (Fig. 3), suggesting that they share a MRCA at node A (bootstrap value: 99%). Because the exterior SIV(Vpx+)/HIV-2 clade have functional Vpx and the interior SIV(Vpx+) sub-clade III have Vpx unable to degrade SAMHD1, we believe that the MRCA at node A should have a *vpx* gene; however, whether the Vpx of SIVs at node A is able to degrade human SAMHD1 cannot be determined. These results suggest that the *vpx* gene was lost during SIV evolution from node A to node B (Fig. 3).

### Tracing the origins of Vpx-positive and -negative SIVs

**Figure 4:**
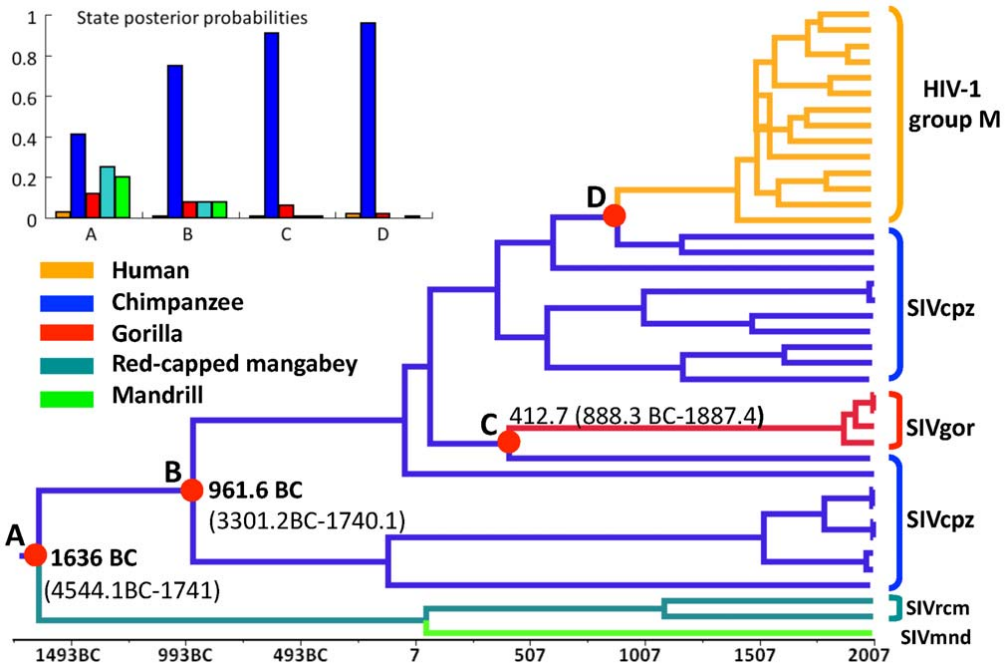
Maximum clade credibility tree of HIV-1 and different SIVs based on their *RT* genes. Ancestral host states were reconstructed using Bayesian phylogeographic inference framework implemented in the BEAST v1.6.2 package. The host state posterior probabilities of the most recent common ancestors (MRCAs) are shown on the left upper panel. The tree branches are colored according to their respective host species. The red solid nodes on the trees represent the MRCAs of corresponding virus strains. Estimated times of the MRCA are shown at corresponding nodes. BC: before Christ.

To better understand the reason why certain SIVs lost or failed to inherit *vpx*, we inferred the ancestral host states of SIVs at nodes A and B. A time scaled maximum clade credibility (MCC) tree was constructed using the sequences that encode reverse transcriptase (RT) from the SIV(Vpx−)/HIV-1 clade and the SIV(Vpx+) sub-clade III (Fig. 4). The MCC tree shows identical topology to the ML tree of *pol* genes (Fig. 3 and 4). The ancestral host states of MRCAs at nodes B, C and D were estimated to be chimpanzees (Fig. 4, left upper panel), indicating that SIVgor and HIV-1 originated by cross-species transmission from chimpanzees to gorillas and humans, respectively. Similarly, the ancestral host state (node A, posterior probability: 0.41) of both the SIV(Vpx−)/HIV-1 clade and the SIV(Vpx+) sub-clade III most likely is chimpanzee (Fig. 4, left upper panel). Moreover, we repeated the analysis using a sequence set including all available 10 SIVrcm and SIVmnd strains. We obtained a higher posterior probability (0.49) to further support that the ancestral host state of MRCA at node A most likely is chimpanzee. These results suggest a cross-species transmission route of an ancestral SIV (Vpx+) from chimpanzees to red-capped mangabeys and/or mandrills, and indicate that the *vpx* gene was lost during SIV evolution among chimpanzees. Further, we estimated the time to the MRCAs (tMRCAs) at several crucial nodes (Fig. 4). The tMRCA (at node B) of the SIV(Vpx−)/HIV-1 clade was estimated at about 2969 (95% highest posterior density (HPD), 267–5308) years ago. The time of the earliest SIV (at node A) infecting chimpanzees was estimated at about 3643 (95% HPD, 266–6551) years ago. Thus, HIV-1 and SIVgor could not inherit the lost *vpx* gene from their ancestor SIVcpz.

### Evolutionary history and loss of the *vpx* gene in certain SIVs and HIV-1

**Figure 5:**
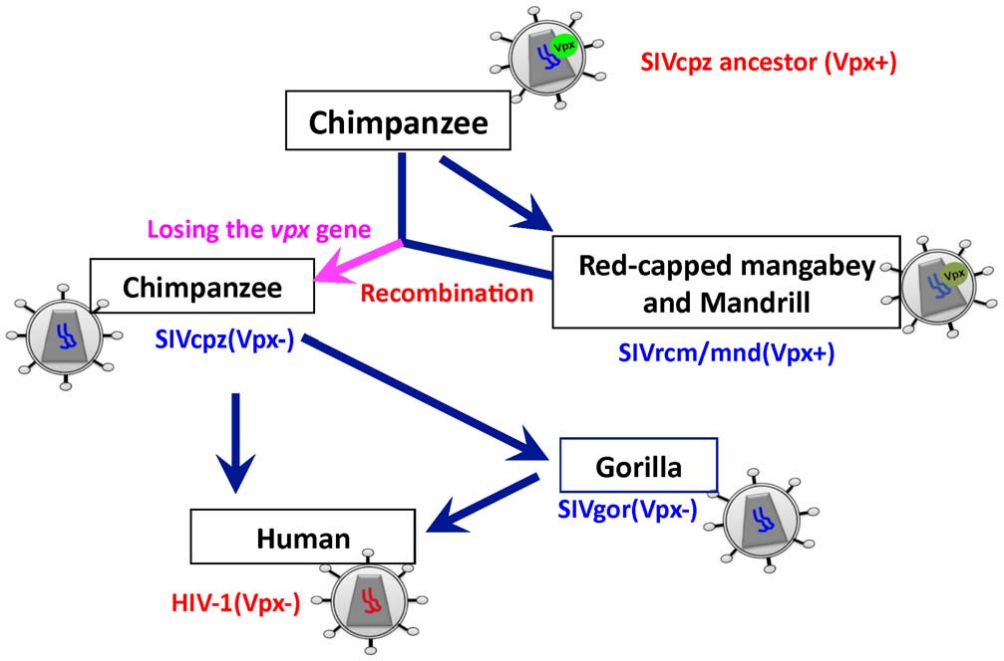
Schematic representation of evolutionary history and loss of the *vpx* gene in certain SIVs and HIV-1. The ancestor virus might have encoded functional Vpx proteins that could degrade SAMHD1. It was likely transmitted to red-capped mangabeys and mandrills prior to the loss of *vpx* gene and evolved into SIVrcm/mnd among the two primate species. Because the ancestral host state of SIVcpz, SIVrcm, SIBmnd, SIVgor and HIV-1 most likely is SIVcpz in the *gag* and *pol* gene regions (Fig. 4, and Fig. S5A), but more likely is SIVrcm or SIVmnd in the *env* region (Fig. S5B), implying that the loss of *vpx* in SIVcpz was a result from positive selection of SIV recombination and viral fitness. The *vpx* gene of SIVcpz was likely lost around 3643 to 2969 years ago, resulting in the emergence of SIVcpz(Vpx−). SIVcpz(Vpx−) was later transmitted to humans and gorillas, evolving into HIV-1 and SIVgor(Vpx−), respectively. The pink arrow indicates the occurrence of *vpx* gene loss.

Our results indicated that an ancestral SIV that had a *vpx* gene and likely infected chimpanzee was inferred to be the MRCA of SIVrcm/mnd(Vpx+), SIVcpz(Vpx−), SIVgor and HIV-1 (Fig. 4). This ancestral SIV lost the *vpx* gene around 3643 to 2969 years ago in chimpanzees and evolved into SIVcpz(Vpx−). Therefore, our results suggest that *vpx* of SIV was lost during evolution among chimpanzees and resulted in the emergence of SIVcpz(Vpx−). After losing *vpx* gene, SIVcpz(Vpx−) likely became the most dominant strains circulating among chimpanzees [19], [20]. Based on these results, we propose a model of evolutionary history and loss of the *vpx* gene in certain SIVs and HIV-1 (Fig. 5). We suggest that, before the occurrence of the *vpx* loss, the ancestral SIVcpz(Vpx+) spread from chimpanzee to red-capped mangabey and mandrill via cross-species transmission, and evolved into SIVrcm(Vpx+) and SIVmnd(Vpx+), respectively (Fig. 5).

## Discussion

A long history of non-human primate infection by SIVs has resulted in a host-virus arms race and led to rapid evolution of both sides [21]. Host restriction factors and their viral antagonists provide an attractive system to investigate genetic conflict between hosts and the viruses. Primate APOBEC3G, TRIM5α and Tetherin undergo positive selection and the positive selection pressures most likely come from the vial antagonists, such as Vif for APOBEC3G, viral capsid for TRIM5α, and Vpu (or SIV Nef) for Tetherin [reviewed in [22]]. HIV-1 genes encoding structural proteins and the polymerase are also under positive selection [23], [24], [25]. Thus, host restriction factors at least partially contribute to the selective pressures on vial antagonists.

SAMHD1 is a myeloid cell-specific HIV-1 restriction factor that can be counteracted by Vpx of SIVsm and HIV-2 [3], [4], while HIV-1 and its ancestor SIVcpz do not encode Vpx to overcome SAMHD1-mediated restriction. It implies that SAMHD1 from human, chimpanzee and gorilla should not be under positive selection, and the lack of *vpx* gene in HIV-1 and its ancestor SIVcpz might be associated with the HIV-1 restriction function of SAMHD1. Indeed, we identified that positive selection mainly acts on SAMHD1 from orangutan, gibbon, rhesus macaque and marmoset, but not on that from human, chimpanzee and gorilla. The lack of selective pressure from Vpx to drive the evolution of SAMHD1 of human, chimpanzee and gorilla is consistent with the fact that HIV-1, SIVcpz, and SIVgor lack the *vpx* gene. Although HIV-2 encodes Vpx and can infect humans, it only accounts for a small population of infected individuals and has a short history (<71 years) in humans [26]. Thus, HIV-2 Vpx is unlikely to be the major driving force behind the evolution of human SAMHD1. The detection of positive selection on the SAMHD1 of other four primates is consistent with the fact that the vast majority of SIV have Vpx, which may drive the rapid evolution of SAMHD1. Our results of positive selection of SAMHD1 confirmed the findings of recent two studies [13], [14].

Virus genes normally have more rapid evolutionary rates than the primate genes [12]. If the primate SAMHD1 is the selective agent, we expect that Vpx that can degrade SAMHD1 should be under positive selection, while Vpx that cannot degrade SAMHD1 should not undergo positive selection. Indeed, we detected positive selection in Vpx from both the HIV-2 sub-clades and the SIV(Vpx+) sub-clades I and II (Table 3), suggesting that Vpx evolves under selective pressure from the primate SAMHD1. Of particular importance is that no positive selection was detected in Vpx of the SIV(Vpx+) sub-clade III, in which Vpx of certain SIVrcm isolates are unable to degrade SAMHD1 [3]. Vpx of two SIVrcm isolates from Nigeria and Gabon cannot degrade human SAMHD1 [3], suggesting that the Vpx of other SIVrcm and SIVmnd are unable to degrade human SAMHD1 due to their high amino acid homology [13] (Fig. S4). Indeed, recent studies demonstrated that Vpx of SIVrcm and SIVmnd can degrade SAMHD1 from red-capped mangabeys and mandrills respectively in a species-specific manner [13], [14].

**Table 3:** Site-model (M7 vs. M8) test for *vpx* genes from two HIV-2 sub-clades and three different SIV(Vpx+) sub-clades.

Sub-clade	dN/dS (M0)	Estimates of parameters		lnL		2Δl	P-value	PSS
		M7	M8	M7	M8			
**HIV-2 sub-clade I**	0.32	p = 0.16255 q = 0.41963	p0 = 0.98103 p = 0.17910 q = 0.48719 (p1 = 0.01897) ω = 4.73	−1925.03	−1915.79	18.49	<0.0001	Yes
**HIV-2 sub-clade II**	0.26	p = 0.11279 q = 0.30495	p0 = 0.94395 p = 0.16311 q = 0.61416 (p1 = 0.05605) ω = 2.22	−1289.45	−1287.91	3.09	0.2133	Yes
**SIV(Vpx+) sub-clade I**	0.17	p = 0.30435 q = 1.41758	p0 = 0.97628 p = 14.36000 q = 99.00000 (p1 = 0.02372) ω = 2.17	−700.66	−700.89	0.47	0.7906	Yes^a^
**SIV(Vpx+) sub-clade II**	0.44	p = 0.13092 q = 0.18331	p0 = 0.94523 p = 40.73726 q = 99.00000 (p1 = 0.05477) ω = 3.79	−700.28	−698.85	2.86	0.2393	Yes
**SIV(Vpx+) sub-clade III**	0.19	p = 0.42080 q = 1.38006	p0 = 0.99999 p = 0.42080 q = 1.38012 (p1 = 0.00001) ω = 1.00	−1666.94	−1666.94	0	1.0000	No

Positively selected sites (PSS) were identified with posterior probability ≥0.90 using Bayes Empirical Bayes (BEB) analysis. M0: one-ratio model.

a One PSS was identified with posterior probability = 0.774 using BEB analysis. When Naive Empirical Bayes analysis was used, the posterior probability = 0.946.

For other details, see Table 1.

A previous study suggested that SIVcpz is a recombinant virus between the predecessor of SIVrcm and the common ancestor of SIVgsn, SIVmus, and SIVmon that infect Greater spot-nosed, Mustached, and Mona monkeys [27]. However, we found that SIVgsn, SIVmus, and SIVmon are located at the basal position in the tree of lentiviruses, far away from the clade containing SIVcpz, implying less possibility of the ancestor of SIVgsn, SIVmus, and SIVmon participating in the recombination. In addition, the inference of the ancestral host states show that the MRCA of SIVcpz, SIVrcm, SIBmnd, SIVgor and HIV-1 most likely is SIVcpz within the *gag* and *pol* gene regions (Fig. 4 and Fig. S5A), but most likely is SIVrcm or SIVmnd in the *env* gene region (Fig. S5B), supporting an association of recombination with the formation of SIVcpz. Therefore, we presume that the loss of *vpx* in SIVcpz was a result from the positive selection of the viral recombination and fitness. Furthermore, the phylogenetic position of Vpx loss have been well documented (reviewed in [28]), which supports our conclusion that co-evolution of primate SAMHD1 and lentivirus Vpx leads to the loss of the *vpx* gene. Of note, the application of HIV molecular clocks to long-term lentivirus evolution has its limitations because heterotachy can cause root ages to be overestimated [29]. Because the sequence data did not include HIV-1 group M sequences obtained after 1996 and SIVgor sequences acquired before 2004, our estimates may not represent the most accurate ones. For example, we dated the origin of HIV-1 group M to 598 (95% HPD, 59–1059) years ago, much earlier than previous estimates based on the *pol* genes (216 years ago, 95% HPD, 111–384) [20].

Our results suggest that HIV-1 could not inherit the lost *vpx* gene from its ancestor SIVcpz (Fig. 5), and the lack of Vpx seems to be advantageous for HIV-1 to escape from human immune surveillance [10], [11], [30]. Thus, the reason for the loss of *vpx* might be associated with the function of chimpanzee SAMHD1. Comparison of amino acid sequences shows that there are 7 different residues between human and chimpanzee SAMHD1 (Fig. S6), including one residue in the SAM domain and three in the HD domain. Whether these differences affect the function of the chimpanzee SAMHD1 to inhibit lentiviral infection need to be determined. Furthermore, SAMHD1 may act as a regulator of the innate immune response [31] and it is unclear whether primate SAMHD1 restricts other viruses in addition to HIV-1. It is also possible that positive selection on primate SAMHD1 has been driven by other pathogens or multiple past host and pathogen conflicts.

In summary, our evolutionary analyses of primate SAMHD1 and lentiviruses provide new insights into understanding the genetic conflict between the restriction factor SAMHD1 and the viral antagonist Vpx. HIV-1 could not inherit the lost *vpx* gene from its ancestor SIVcpz. The lack of Vpx in HIV-1 results in restricted infection in myeloid cells that are important for antiviral immunity, which could contribute to the AIDS pandemic by escaping the immune responses.

## Materials and Methods

### Primate SAMHD1 sequences from database

The human SAMHD1 gene was retrieved from GenBank. BLAST searches were performed in GenBank and Ensembl genome assemblies using human SAMHD1 gene. The E value <0.001 and the presence of both SAM and HD domains were used to determine whether a SAMHD1 sequence was found. To gain a full list of SAMHD1 genes, several iterations of searches were performed using each newly obtained SAMHD1 sequence as a query. As a result, six primate sequences were obtained, including human (*Homo sapiens*, AAH36450.1), gorilla (*Gorilla gorilla*, ENSGGOG00000011336), orangutan (*Pongo abelii*, XP_002830320.1), gibbon (*Nomascus leucogenys*, XP_003253588.1), rhesus macaque (*Macaca mulatta*, XP_001097562.2) and Marmoset (*Callithrix jacchus*, XP_002747259.1).

### Sequences of chimpanzee SAMHD1 cDNA

Because incomplete genomic sequencing of chimpanzee (*Pan troglodytes*) that leads to two gaps in the SAMHD1 coding sequence (XP_514624.3), we amplified and sequenced chimpanzee SAMHD1 cDNA. Two chimpanzee B cell lines and cDNA sample derived from chimpanzee liver [32], [33] were kind gifts from Dr. Barbara Rehermann (National Institutes of Health). Total RNA from the lymph node of a chimpanzee [34] was obtained through Dr. Robert Palermo (University of Washington). The collection of these chimpanzee samples has been initially approved by the Institutional Animal Care and Use Committees (IACUC) of the National Institutes of Health and the University of Washington, respectively. The use of chimpanzee cells and RNA samples has been approved by the IACUC of The Ohio State University (protocol 2011A00000113). RNA samples from three chimpanzees were used to amplify chimpanzee SAMHD1 cDNA sequences by RT-PCR (Platinum Taq DNA Polymerase High Fidelity, Invitrogen), and the PCR products were directly sequenced. Primer sequences used in the PCR amplification of chimpanzee SAMHD1 cDNA are as follows: forward – 5′ GAC TGC TGT GCC GGA CG; reverse – 5′ CAT TGG GTC ATC TTT AAA AAG CTG GAC TC. GenBank accession numbers are JQ085409, JQ085410, and JQ085411 for SAMHD1 cDNA sequences derived from the liver, lymph, and B cells, respectively. The sequences from the lymph tissue and B cells are identical to each other with a single amino acid difference from that of the liver tissue. Only the sequence from the liver tissue was used in subsequent analyses.

### The primate lentivirus sequences

All available near full length genomic sequences of SIV and HIV-2 were retrieved from the HIV sequence databases (http://www.hiv.lanl.gov/content/index). After deleting identical sequences, 124 SIV and 36 HIV-2 genomic sequences were obtained. Because of too many full-length genomic sequences of HIV-1 available in HIV-1 database, only 2008 subtype reference of HIV-1 group M excluding recombinants were retrieved. The genomic sequences of SIV, HIV-1 and -2 were initially aligned using Muscle implemented in MEGA5.03 [35]. Then, the *pol* and *vpx* gene sequences were respectively cut out from the three genomic sequence sets. By merging the target sequences from SIV, HIV-1 and HIV-2, and deleting identical sequences, a total of 182 *pol* sequences were kept for subsequent analyses. Because HIV-1 and some SIVs lack the *vpx* gene, only 61 *vpx* sequences were kept for further analyses.

### Phylogenetic analyses of primate SAMHD1 sequences

A complete alignment of seven primate SAMHD1 protein-coding sequences was obtained using webPRANK (http://www.ebi.ac.uk/goldman-srv/webPRANK/). Based on this alignment, Bayesian and maximum likelihood (ML) phylogenetic trees were constructed using mrbayes-3.1.2 [36]and MEGA 5.03, respectively. In Bayesian analysis, four independent Markov Chain Monte Carlo (MCMC) chains were used with the default temperature of 0.1. Four repetitions were run for 50000 generations with tree and parameter sampling occurring every 10 generations. If the standard deviation of split frequencies is below 0.01 after 50,000 generations, the run was stopped. The first 25% of trees were discarded as burn-in, leaving 3750 trees per run. Posterior probabilities for internal node were calculated from the posterior density of trees. The ML analysis was performed with the best-fitting nucleotide substitution model of HKY+I+G and a bootstrap analysis of 1,000 replications.

The numbers of non-synonymous substitutions per nonsynonymous site (dN) and that of synonymous nucleotide substitutions per synonymous site (dS) were estimated by the modified Nei–Gojobori method implemented in MEGA 4.0 with an estimated transition/transversion ratio of 1.679 [37]. According to the phylogeny of the seven primates analyzed, the ancestral SAMHD1 sequence at each interior node of the ML tree was inferred with the Anc-gene software [38], and then the numbers of synonymous (s) and non-synonymous (n) substitutions on each branch were counted. In addition, the free-ratio model in PAML 4.2 package that allows ω (dN/dS) to vary along each branch was used to assume an independent ω value for each branch of the tree [39]. Hon-new software was used to evaluate the radical and conservative non-synonymous substitutions with regard to amino acid charge, polarity, and polarity and volume [40].

The site-specific model was performed to further detect positive selection on individual sites using the program codeML in PAML 4.2 package. Three selective models 2a, 3 and 8 that allow for positive selection (ω>1) were compared with three null models 0, 1a and 7 that do not allow for positive selection, respectively. Likelihood ratio test was used to determine the significance of difference between the null model and the alternative model by calculating twice the log-likelihood difference following a χ^2^ distribution, with the number of degrees of freedom. The Bayes empirical Bayes approach in M2a and M8 was used to determine PSS [41]. The sites with ω>1 and posterior probabilities of ≥0.90 were identified as PSS.

### Phylogenetic analyses of viral *pol* and *vpx* sequences

After realignment of the *pol* and *vpx* sequences using Muscle, ML phylogenetic trees of *pol* and *vpx* sequences were reconstructed using MEGA 5.03 with the best-fitting nucleotide substitution models of CTR+G+I and K2+G, respectively. The bootstrap analyses were performed with 100 replications. In *vpx* tree, HIV-2 and SIV vpx were divided into 2 and 3 sub-clades, respectively. These sub-clades were also subjected to the site-specific analyses as described above.

### Reconstruction of time scale and ancestral host states of SIVcpz, SIVrcm/mnd, SIVgor and HIV-1

To trace the plausible diffusion routes of SIV between red-capped mangabey/mandrill, chimpanzee and gorilla, the *pol* gene sequences from SIVcpz, SIVrcm/mnd, SIVgor and HIV-1 were used to construct a time scaled MCC tree with a MCMC method implemented in the BEAST v1.6.2 package [42]. Each sequence was assigned two characters reflecting its sampling time and host status using BEAUti v1.6.2. The evolutionary rates and the times to the most recent common ancestors (tMRCA) of various nodes in the MCC tree were estimated by BEAST. The GTR+G+I nucleotide substitution model, the uncorrelated log-normal relaxed clock model and the constant population size coalescent tree prior were used in the MCMC analyses. Statistical uncertainty in parameter estimates was given by the values of the 95% highest posterior density. Each MCMC analysis was run for 200 million generations, with sampling every 10,000 generations. The initial 25% of the trees were discarded as burn-in when we summarized the trees using TreeAnnotator v1.6.2. The program Tracer v1.5 (tree.bio.ed.ac.uk/software/tracer/) was used to check for the convergence (effective sample size >200) with 10% burn-in.

Ancestral host states were inferred using a geographically explicit Bayesian MCMC method under the asymmetric CTMC model for discrete state reconstructions implemented in the BEAST v1.6.2 package [43], [44]. This method can be used to infer the host state of the ancestral branch over the whole tree and to build a reversible diffusion rate matrix between previously defined host species accompanied with the evolutionary and coalescent parameters set above. The ancestral host origins were evaluated by posterior probability that is calculated from the posterior density of trees.

## Supporting Information

Figure S1 **Pairwise comparisons of dN and dS among seven primate SAMHD1 sequences.** The red arrow indicates the data point with dN/dS>1.
(TIF)

Figure S2 **The amino acid mutations fixed by orangutan SAMHD1 sequence.** The green shadows indicate the amino acid mutations were detected to be under positive selection by PAML 4.2. The SAM and HD domains are highlighted by the blue and pink frames, respectively. The sequences corresponding to the gap sequences in rhesus macaque and marmoset SAMHD1 (see Fig. 5) were excluded from the analysis.
(TIF)

Figure S3 **Maximum likelihood tree of** ***vpx*** **genes from SIV and HIV-2.** SIVcpz/gor and HIV-1 group M that lost the *vpx* genes were merged into the tree based on the topology of *pol* gene tree (Figure 3). Because the Vpx of two SIVrcm isolates from Nigeria and Gabon cannot degrade human SAMHD1 [3], we predicted that the Vpx from other SIV strains in SIV(Vpx+) sub-clade III may not be able to degrade human SAMHD1. The red solid nodes on the trees represent the most recent common ancestors (MRCAs) of corresponding virus strains. The thick pink branch indicates the occurrence of *vpx* gene loss.
(TIF)

Figure S4 **Protein sequence logo of Vpx from five SIV sub-clades.** The Vpx amino acid sequence characteristic of five SIV sub-clades were generated using WebLogo (http://weblogo.threeplusone.com/create.cgi). The overall height of the stack indicates the sequence conservation at that position and the height of each symbol within the stack indicates the relative frequency of an amino acid at that position. The red triangles indicate two conserved sites of SIV Vpx (Q76 and F80), which are crucial for Vpx-mediated degradation of human SAMHD1 [3], [4].
(TIF)

Figure S5 **Maximum clade credibility tree of HIV-1 and different SIVs based on their** ***gag*** **(A) and** ***env*** **(B) genes.** Analyzed *gag* and *env* sequences correspond to the nucleotides 796–1542 and 7776–8459 in HIV-1 HXB2 genome, respectively. For more details, please see Figure 4 and Figure 4 legend.
(TIF)

Figure S6 **Alignment of the amino acid sequences of SAMHD1 from seven primates.** The black small dots indicate identity to the human sequence and dash indicates a gap. Red solid circles, triangles, and squares indicate the PSS with posterior probabilities (p) of ≥0.90, 0.95, and 0.99, respectively. The green and pink shadows indicate the SAM and HD domains, respectively. The SAMHD1 sequence from chimpanzee liver tissue was determined in this study and all other sequences were retrieved from GenBank or Ensembl database.
(TIF)
