# Supplementary figures and images for: Co-Evolution of Primate SAMHD1 and Lentivirus Vpx Leads to the Loss of the vpx Gene in HIV-1 Ancestor
Source: PLoS One. 2012 May 4;7(5):e37477. doi: 10.1371/journal.pone.0037477 (PMC3345027; doi:10.1371/journal.pone.0037477)

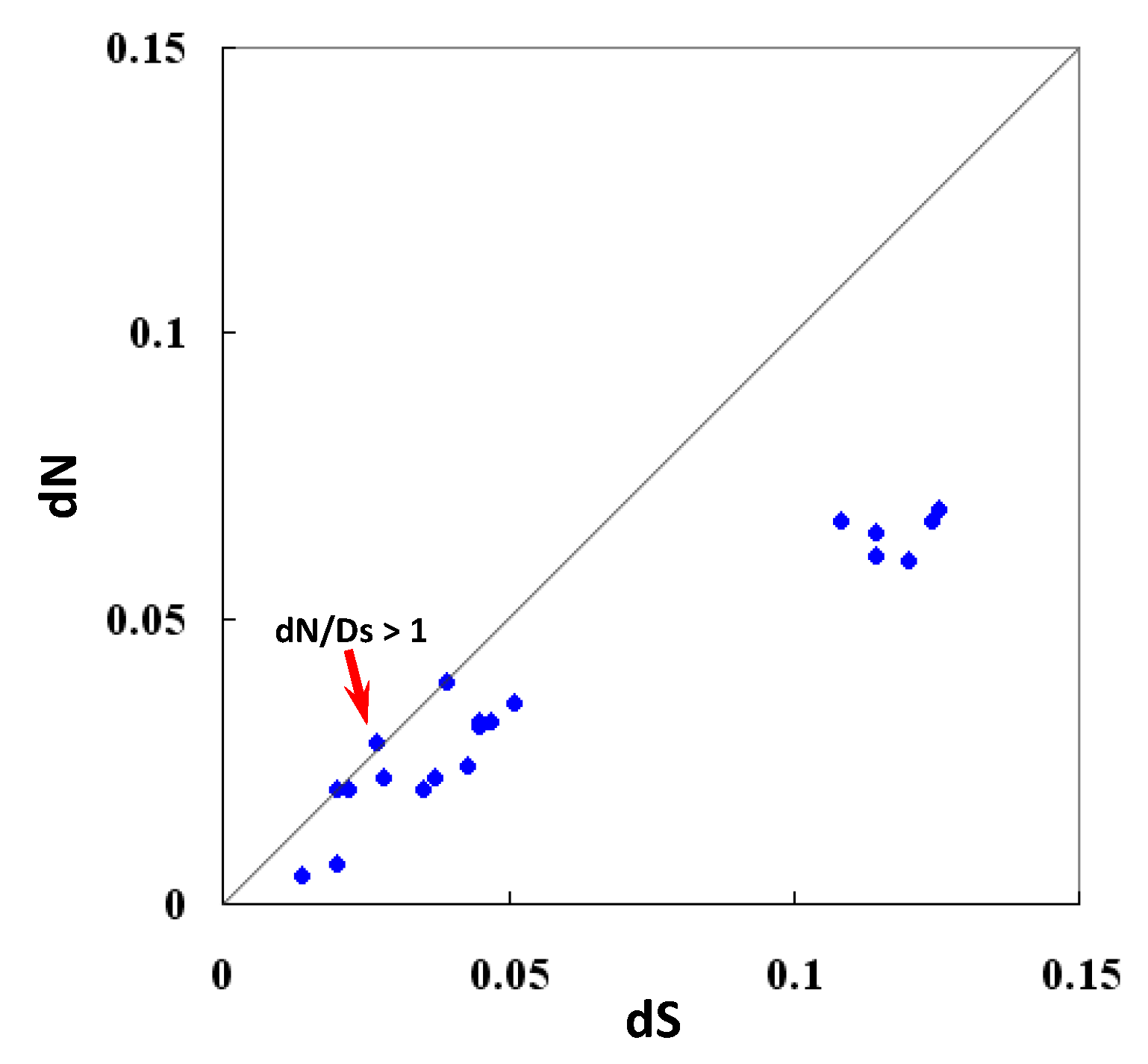

Supplement: Figure S1 — Pairwise comparisons of dN and dS among seven primate SAMHD1 sequences. The red arrow indicates the data point with dN/dS>1. (TIF) [file pone.0037477.s001.tif]

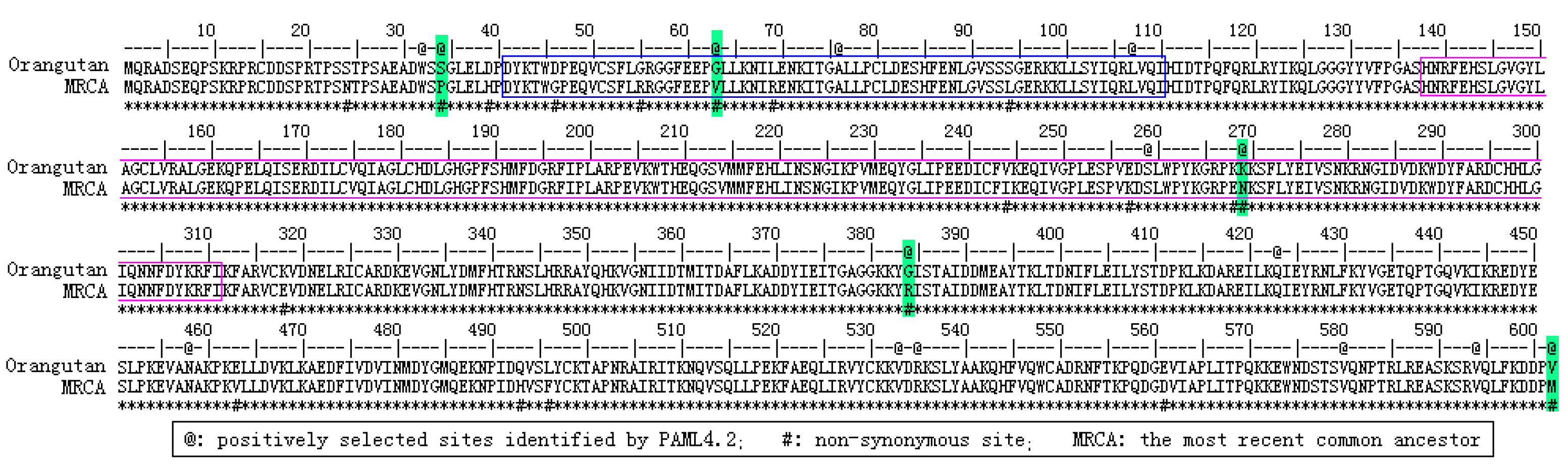

Supplement: Figure S2 — The amino acid mutations fixed by orangutan SAMHD1 sequence. The green shadows indicate the amino acid mutations were detected to be under positive selection by PAML 4.2. The SAM and HD domains are highlighted by the blue and pink frames, respectively. The sequences corresponding to the gap sequences in rhesus macaque and marmoset SAMHD1 (see Fig. 5) were excluded from the analysis. (TIF) [file pone.0037477.s002.tif]

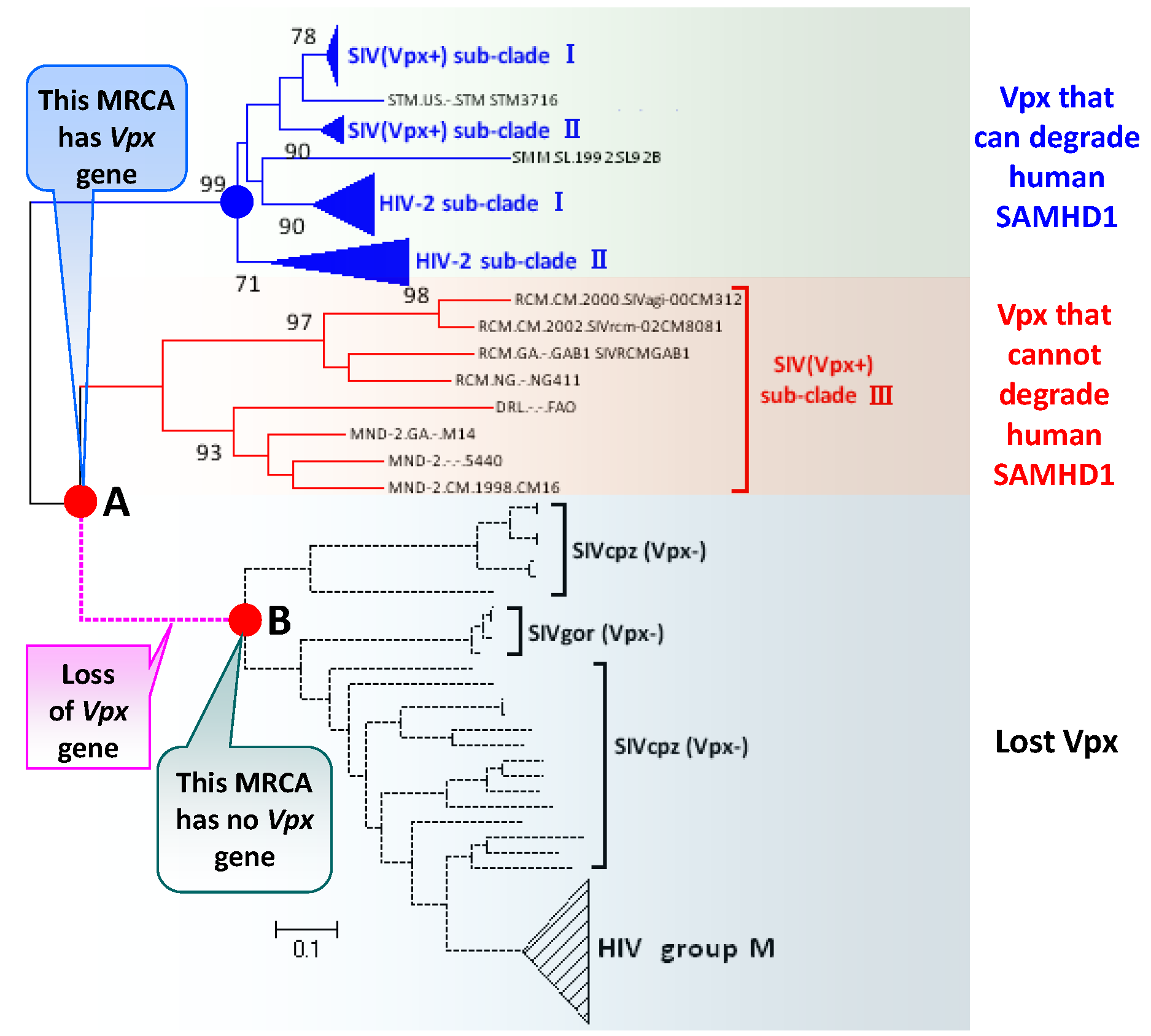

Supplement: Figure S3 — Maximum likelihood tree of vpx genes from SIV and HIV-2. SIVcpz/gor and HIV-1 group M that lost the vpx genes were merged into the tree based on the topology of pol gene tree (Figure 3). Because the Vpx of two SIVrcm isolates from Nigeria and Gabon cannot degrade human SAMHD1 [3], we predicted that the Vpx from other SIV strains in SIV(Vpx+) sub-clade III may not be able to degrade human SAMHD1. The red solid nodes on the trees represent the most recent common ancestors (MRCAs) of corresponding virus strains. The thick pink branch indicates the occurrence of vpx gene loss. (TIF) [file pone.0037477.s003.tif]

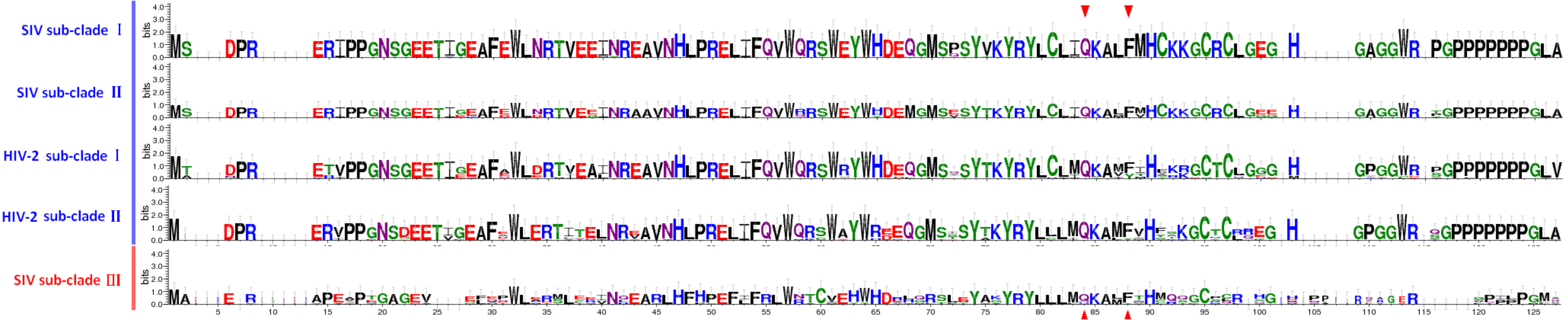

Supplement: Figure S4 — Protein sequence logo of Vpx from five SIV sub-clades. The Vpx amino acid sequence characteristic of five SIV sub-clades were generated using WebLogo (http://weblogo.threeplusone.com/create.cgi). The overall height of the stack indicates the sequence conservation at that position and the height of each symbol within the stack indicates the relative frequency of an amino acid at that position. The red triangles indicate two conserved sites of SIV Vpx (Q76 and F80), which are crucial for Vpx-mediated degradation of human SAMHD1 [3], [4]. (TIF) [file pone.0037477.s004.tif]

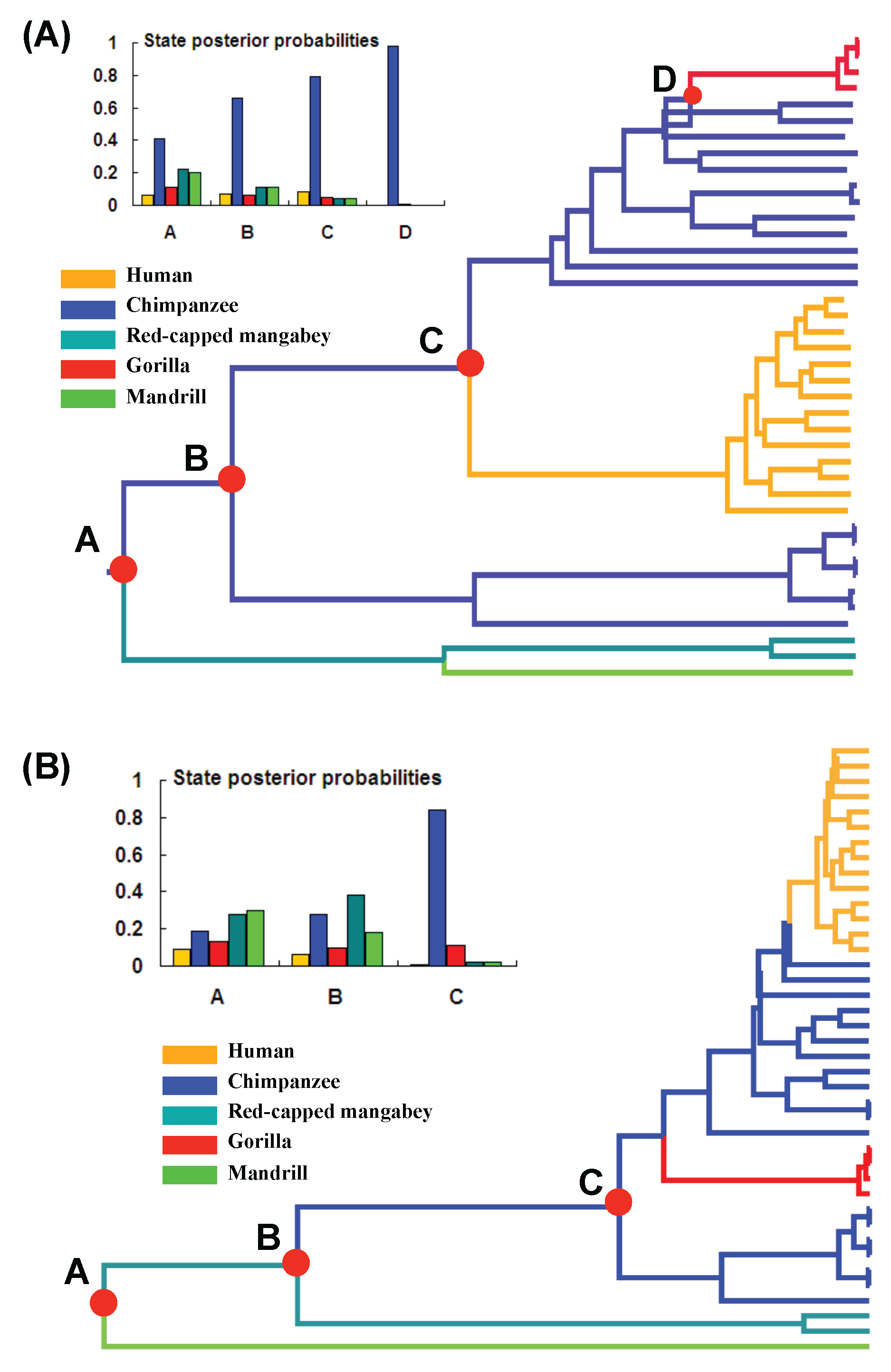

Supplement: Figure S5 — Maximum clade credibility tree of HIV-1 and different SIVs based on their gag (A) and env (B) genes. Analyzed gag and env sequences correspond to the nucleotides 796–1542 and 7776–8459 in HIV-1 HXB2 genome, respectively. For more details, please see Figure 4 and Figure 4 legend. (TIF) [file pone.0037477.s005.tif]

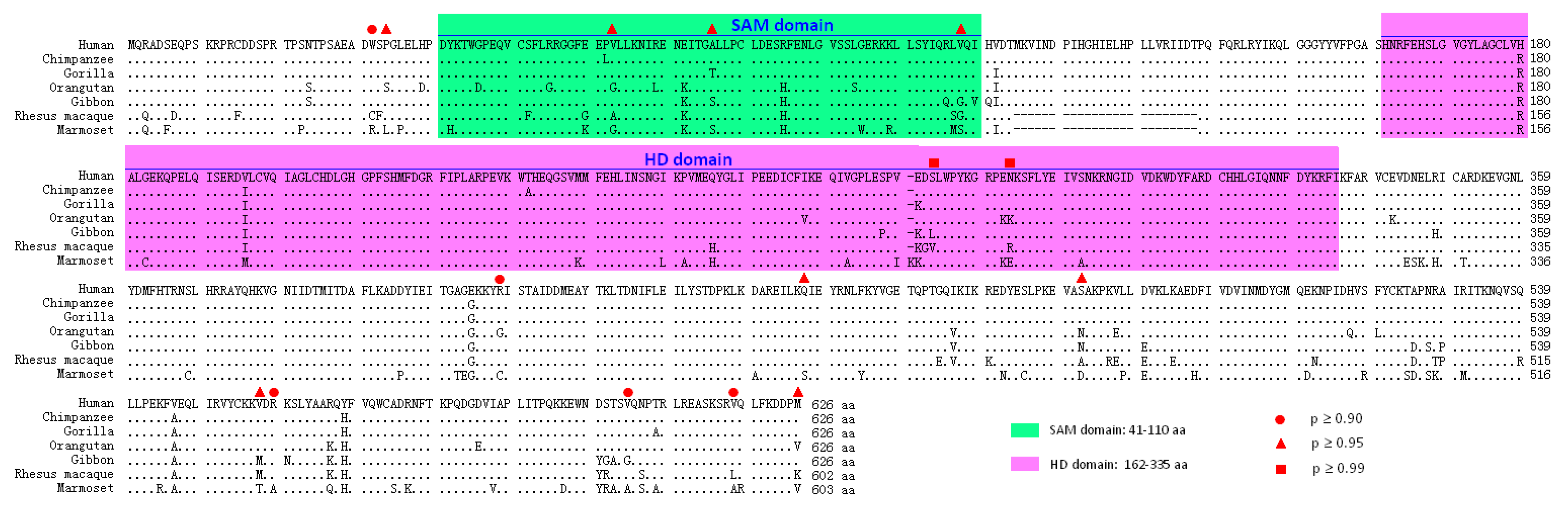

Supplement: Figure S6 — Alignment of the amino acid sequences of SAMHD1 from seven primates. The black small dots indicate identity to the human sequence and dash indicates a gap. Red solid circles, triangles, and squares indicate the PSS with posterior probabilities (p) of ≥0.90, 0.95, and 0.99, respectively. The green and pink shadows indicate the SAM and HD domains, respectively. The SAMHD1 sequence from chimpanzee liver tissue was determined in this study and all other sequences were retrieved from GenBank or Ensembl database. (TIF) [file pone.0037477.s006.tif]
